# Development and application of patient-reported experience measures for cancer patients: a scoping review

**DOI:** 10.1016/j.ijnsa.2025.100327

**Published:** 2025-04-11

**Authors:** Qiongjie Shao, Wei Zhang, Hongjuan Lang, Yan Wang, Han Tang, Juan Du, Ying Liang, Pengyu Jing, Zhongping Gu, Xiaolong Yan, Lei Shang

**Affiliations:** a Department of Health Statistics, School of Public Health, Fourth Military Medical University, Xi'an, Shaanxi, PR China; b Department of Thoracic Surgery, Tangdu Hospital, Fourth Military Medical University, Xi'an, 710038, Shaanxi, PR China; c Department of Clinical Nursing, School of Nursing, Fourth Military Medical University, Xi'an, Shaanxi, PR China

**Keywords:** Experience, Measures, Cancer, Patients, Patient-centered care, Scoping review, Tool

## Abstract

**Objective:**

This study examines the currently available Patient-Reported Experience Measures for cancer patients and provides a scoping overview of their definitions, evaluation frameworks, assessment tools, and current applications. The findings aim to inform and guide the development of a patient-centered care model.

**Methods:**

Using a combination of subject terms and free-text keywords, studies published by national and international initiatives were reviewed across three online databases (PubMed, Web of Science, and MEDLINE) following the PRISMA guidelines.

**Results:**

A total of 2216 papers were reviewed, of which 24 were included in the scoping review. From these, 11 Patient-Reported Experience Measures were identified, each from 10 different national projects. Definitions of Patient-Reported Experience Measures were established, and the current status of Patient-Reported Experience Measures evaluation systems for cancer patients, along with their application across four domains, was analyzed.

**Conclusion:**

Currently, research on Patient-Reported Experience Measures in cancer patients remains in its early stages, and the effectiveness of several assessment tools has yet to be fully validated. Future studies should focus on developing high-quality, cancer-specific Patient-Reported Experience Measures assessment tools. These tools should be rigorously evaluated and tailored to the unique characteristics of cancer patients' healthcare experiences, with the aim of supporting and enhancing patient-centered care practices.

## 1 Introduction

The global cancer burden has been steadily rising in recent years, primarily due to an aging population and increased exposure to risk factors (Romero et al., 2025). According to the latest global cancer statistics released by the International Agency for Research on Cancer (IARC), approximately 20 million new cases of malignant tumors were reported worldwide in 2022, resulting in around 9.7 million fatalities. IARC projects that by 2050, the global incidence of new cancer cases will exceed 35 million, representing a 77 % increase from 2022 levels (Han et al., 2024; Global cancer burden growing, amidst mounting need for services, 2024). These statistics highlight the significant challenges facing cancer prevention and treatment efforts.

Currently, traditional surgical resection remains a fundamental approach to treating malignant tumors, while radiotherapy, chemotherapy, molecular targeted therapy, and immunotherapy play crucial roles in cancer treatment. In recent years, guidelines from the National Comprehensive Cancer Network (NCCN) and the American Society of Clinical Oncology (ASCO) have integrated evidence-based medicine and the latest advances in precision medicine, offering clinicians standardized recommendations for the diagnosis and treatment of malignant tumors (Roeland et al., 2023; Zhu et al., 2023). However, these diagnostic and treatment tools often prioritize clinical judgment and therapeutic outcomes, sometimes at the expense of considering patients' needs and preferences.

With the evolution of healthcare models and the continuous improvement of patients' awareness and health literacy, there is a growing recognition that enhancing the quality of medical services should prioritize patients' needs and experiences (Mielke et al., 2024). This shift has established the people-centered healthcare model as a vital criterion for evaluating healthcare quality globally. "Patient-centeredness" embodies this philosophy in the medical field, focusing on respecting and fulfilling patients' needs, preferences, and values while ensuring these crucial factors are fully integrated into clinical decision-making. In hospitals, nursing staff play a central role in delivering patient-centered services. Patient-centered care (PCC) encompasses five key elements: collaborative treatment, the biopsychosocial model, compassionate care approaches, shared decision-making and responsibility, and coordinated nursing services (Havana et al., 2023). Positive patient experiences are closely associated with improved disease prevention, advanced diagnostic and treatment methods, better clinical outcomes, a strengthened patient safety culture, and more convenient healthcare processes.

Patient-reported measures refer to reports provided directly by patients about their health status, health behaviors, or experiences receiving medical care, without the need for interpretation by healthcare professionals or other intermediaries (Minvielle et al., 2023). Patient-reported measures are divided into two main types: Patient-reported Experience Measures and Patient-reported Outcome Measures (Howard et al., 2024). Patient-Reported Experience Measures are widely used in developed countries to assess patient-centered care processes through standardized tools. This helps healthcare institutions understand and improves service quality, ultimately enhancing patient satisfaction. Patient-Reported Experience Measures are designed to document the full healthcare experience from the patient's perspective using objective measurement tools.

Since 1990s, Patient-reported Experience Measures have attracted significant interest from international scholars, with numerous publications discussing their underlying concepts. Although the idea of Patient-Reported Experience Measures was first introduced by American academic Harvey Picker (Frampton and Guastello, 2008) in 1986, a precise definition has yet to be established. The Beryl Institute(Wolf, no date) in the United States proposed a widely accepted concept of Patient-Reported Experience Measures, defining them as measures of all patient-perceived interactive processes during consultations and treatments, influenced by organizational culture. According to American scholar Bull, Patient-Reported Experience Measures serve as an assessment tool that captures, from the patient's perspective, what occurs and which type of care is delivered (Bull et al., 2019). The National Health Service (NHS) in the United Kingdom has delineated the primary components of Patient-Reported Experience Measures. These components include respect for the patient, coordination and integration of services, effective communication of information and health education, physical comfort, emotional support, encouragement of family and friend involvement, transitions and continuity of services, and accessibility of services (Ahmed et al., 2014). Patient-Reported Experience Measures assess how patients perceive various aspects of the healthcare experience, including waiting times for appointments, organizational features (such as communication from healthcare providers), emotional responses (like concerns about pain), and the overall care process. While specific Patient-Reported Experience Measures are designed for patients with particular conditions, generic Patient-Reported Experience Measures are applicable to all patient populations.

In recent studies, Patient-Reported Experience Measures have enhanced cancer treatment tolerance, reduced hospital stays and emergency room visits, facilitated early detection of metastasis and recurrence, and significantly improved cancer patients' survival rates and quality of life (Saunders et al., 2016). However, there is a lack of comprehensive searches for available Patient-Reported Experience Measures assessment tools, and standardized Patient-Reported Experience Measures research specific to cancer patients is still in its infancy. Consequently, this study conducts a scoping review to explore the definition, evaluation system, assessment tools, and current application status of Patient-Reported Experience Measures in the clinical diagnosis and treatment of cancer patients, aiming to achieve a more comprehensive and objective understanding of this field. We will provide valuable references for the development and application of Patient-Reported Experience Measures assessment tools and - lay a solid foundation for establishing a patient-centered care model. The core questions we declaim in this review: (1) What is the definition of Patient-Reported Experience Measures? (2) What Patient-Reported Experience Measures assessment instruments are available for cancer patients? (3) How is the Patient-Reported Experience Measures assessment system structured for cancer patients? (4) What is the current status of Patient-Reported Experience Measures use in cancer patients?

## 2 Methods

This review is publicly registered on the Open Science Framework (OSF) with registration number 10.17605/OSF.IO/RVTQG and follows the PRISMA extension for scoping reviews (PRISMA-ScR) criteria (McGowan et al., 2020).

### 2.1 Literature selection

Inclusion Criteria: (1) Cancer patients aged≥18 years; (2) Studies focused on the development or implementation of Patient-Reported Experience Measures assessment methods for cancer patients; (3) Full studies published in English in peer-reviewed journals.

Exclusion criteria: (1) Literature types such as reviews, editorials, commentaries, and conference abstracts; (2) Studies focused on patient satisfaction, quality of care, attitude scales, or Patient-reported Outcome Measures; (3) Studies specifically related to patient experience with a particular treatment or intervention; (4) Studies in which proxies, rather than patients, completed the Patient-Reported Experience Measures.

### 2.2 Search strategy

**Fig. 1:**
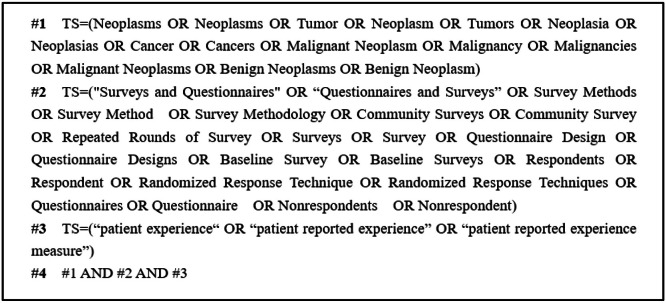
Web of Science search strategies.

To ensure comprehensive identification of relevant studies, we conducted searches not only in electronic databases but also through the reference lists of all retrieved articles. Additionally, a manual search was performed using Google Scholar. Search terms included combinations of 'cancer, oncology, malignancy, neoplasms'; 'patient experience, patient-reported experience, patient-reported experience measure'; and 'measure, tool, instrument, score, scale, survey, questionnaire, psychometrics.' The English search terms 'reported experience measure' and 'measure, tool, instrument, score, scale, survey, questionnaire, psychometrics' were used in PubMed, Web of Science, and MEDLINE databases, employing a combination of subject terms and free-text words. The search covered the period from the inception of each database until July 2024. Fig. 1 illustrates the detailed search strategy.

### 2.3 Literature screening and data extraction

This study utilized Zotero software to manage the literature and Microsoft Excel to extract key information from the included studies. Two researchers independently followed the inclusion criteria, first screening the titles and abstracts, then reviewing the full texts of any studies that potentially met the criteria. In case of disagreement, a third reviewer was consulted and made the final decision to resolve any conflicts. Data extracted from the included studies included author information, year of publication, country, study type, study population, sample size, Patient-Reported Experience Measures assessment tool, type of Patient-Reported Experience Measures, reporting method, evaluation metrics, content of the Patient-Reported Experience Measures assessment, study limitations, future perspectives, and other relevant details. These data were then summarized and analyzed.

### 2.4 Literature quality evaluation

All manuscripts included in this review were assessed for quality using the Quality Assessment for Diverse Studies (QuADS) checklist (Harrison et al., 2021). We selected the QuADS checklist due to its broad applicability to the diverse types of studies included in the review. The quality ratings for each study are presented in Table 1.

**Table 1:** Quality ratings of included studies.

Study (Reference)	Item 1	Item 2	Item 3	Item 4	Item 5	Item 6	Item 7	Item 8	Item 9	Item 10	Item 11	Item 12	Item 13
(Alessy et al., 2019)	2	3	3	2	3	1	3	3	2	1	3	2	3
(Arditi et al., 2023)	2	3	3	2	2	1	3	3	2	3	3	3	2
(Brookes and Baker, 2022)	3	3	2	3	2	1	3	1	2	3	3	3	2
(Cha et al., 2022)	1	2	2	2	2	1	2	1	1	2	2	1	3
(Christalle et al., 2022)	3	3	2	3	3	2	3	3	0	2	2	3	0
(Clucas, 2016)	2	3	2	2	2	2	2	1	2	0	2	1	2
(Fauer et al., 2021)	1	2	1	2	1	2	2	1	2	1	2	0	2
(Fernstrom et al., 2016)	2	3	2	2	2	3	2	2	1	3	3	3	3
(Gomez-Cano et al., 2022)	1	2	3	2	2	1	3	1	1	3	3	2	3
(Iversen et al., 2012)	2	3	3	3	3	3	3	2	3	3	2	2	1
(Karabatić et al., 2022)	3	3	2	2	2	2	3	1	1	0	0	2	1
(Moens et al., 2022)	3	3	1	3	0	3	3	3	0	3	3	3	2
(Nartey et al., 2022)	2	2	3	3	2	2	2	2	1	2	3	2	2
(Pham et al., 2019)	0	2	3	2	2	1	2	1	2	1	2	0	2
(Reid et al., 2023)	3	3	3	3	2	3	3	3	2	3	3	2	3
(Roth et al., 2020)	2	3	3	2	2	3	2	3	3	1	2	0	2
(Rudolph et al., 2019)	2	3	3	2	2	3	3	3	2	1	2	3	3
(Saunders et al., 2014)	0	2	2	2	2	2	2	3	2	3	3	2	2
(Taibi et al., 2023)	0	2	1	2	0	2	2	1	1	2	2	2	2
(Wagland et al., 2017)	1	2	3	3	2	2	2	2	1	3	2	2	2
(Watanabe et al., 2021)	1	1	3	2	3	3	3	2	3	2	2	3	2
(Yu et al., 2018)	2	3	3	2	2	2	3	3	1	2	2	3	3
(Shah et al., 2024)	0	3	3	2	2	2	2	3	1	2	2	1	3
(van Hof et al., 2024)	2	3	2	3	0	3	0	0	0	2	2	1	3

**Notes:** For item 1. Theoretical or conceptual underpinning to the research, the grading criteria is:.

0: No mention Theoretical or conceptual underpinning to the research or prior studies related to the research;.

1: General reference to broad theories or concepts that frame the study or mention related prior studies;.

2: Identification of specific theories or concepts that frame the study and how these informed the work undertaken or mention closely related prior studies;.

3: Explicit discussion of the theories or concepts that inform the study, with application of the theory or concept evident through the design, materials and outcomes explored or mention closely related prior studies and analyze limitations and improve these limitations in the research.

## 3 Results

### 3.1 Search results

**Fig. 2:**
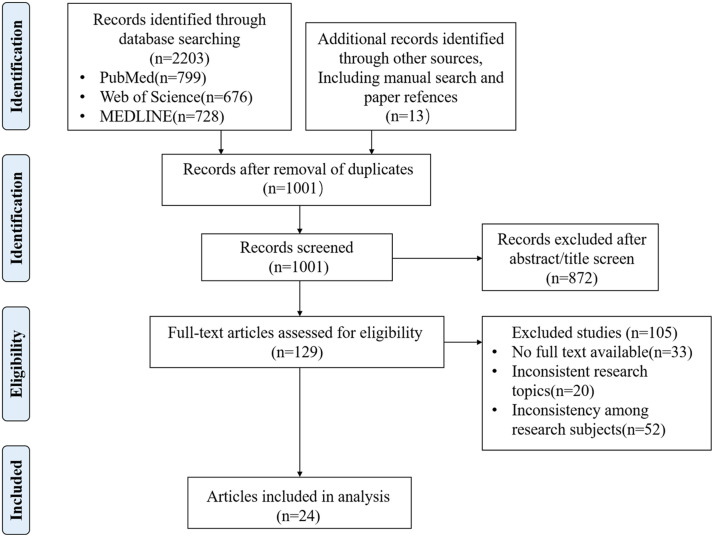
PRISMA flow diagram.

A total of 2,203 records were initially identified, with an additional 13 documents sourced from other references. After removing duplicates, 1,001 unique records remained. Of these, 872 records were excluded based on title and abstract screening, and 105 were further excluded after full-text review. Ultimately, 24 studies written in English were included in the final analysis (Fig. 2).

### 3.2 Study characteristics

A total of 24 papers, published between 2012 and 2024, were reviewed. The studies originated from the United Kingdom (*n* = 9), United States (*n* = 4), Germany (*n* = 2), Netherlands (*n* = 1), Norway (*n* = 1), Croatia (*n* = 1), Belgium (*n* = 1), Australia (*n* = 1), France (*n* = 1), Japan (*n* = 1), Taiwan (*n* = 1), and Switzerland (*n* = 1). The largest sample size included was 214,340 cases. The key characteristics of the included studies are summarized in Table 2.

**Table 2:** Study characteristics (*n* = 24).

Author	Year	Country	Population	Sample size	Assessment tool	Applicability	Primary objective
(Alessy et al., 2019)	2019	UK	Patients with colorectal, lung, breast, and prostate cancer	103,186	National Cancer Patient Experience Survey (NCPES)	all cancer types	Directing the creation, oversight, and enhancement of cancer policy
(Arditi et al., 2023)	2023	Switzerland	Patients with hematological cancer, colorectal, lung, breast, prostate, or colorectal	2696	Swiss Cancer Patient Experiences (SCAPE)	all cancer types	Recognize the differences in Patient-reported Experiences between patients with various characteristics and focus efforts on improving.
(Brookes and Baker, 2022)	2022	UK	cancer patients	214,340	National Cancer Patient Experience Survey (NCPES)	all cancer types	Recognizing the percentage of patients who provide feedback regarding both positive and bad experiences, as well as response themes that inform assessment
(Cha et al., 2022)	2022	USA	cancer patients	2868	Hospital Consumer Assessment of Healthcare Providers and Systems (HCAHPS)	all cancer types	Provide crucial data to oncologists and clinical practitioners, as well as for the creation of national health policies.
(Christalle et al., 2022)	2022	Germany	Patients with Cancer, cardiovascular disease, mental illness, or musculoskeletal disorders	2000	Experienced Patient-Centeredness Questionnaire (EPAT)	chronic diseases	Helpful in evaluating components of quality improvement in routine clinical inpatient and outpatient investigations.
(Clucas, 2016)	2016	UK	cancer patients	45,191	National Cancer Patient Experience Survey (NCPES)	all cancer types	Help explain disparities in experience between patient groups and provide more tailored interventions.
(Fauer et al., 2021)	2021	USA	elderly patients with lymphoma or leukemia diagnoses	1151	Hospital Consumer Assessment of Healthcare Providers and Systems (HCAHPS)	all cancer types	Enhancements made to patients' treatment phases in response to Patient-reported Experience data to guide clinical practice
(Fernstrom et al., 2016)	2016	USA	Patients with heart failure, cancer, or dementia	903	LifeCourse experience tool	patients with chronic life-limiting illnesses	Contribute to developing an evidence base to drive the growth of clinical practice standards.
(Gomez-Cano et al., 2022)	2022	UK	cancer patients	71,186	National Cancer Patient Experience Survey (NCPES)	all cancer types	Able to serve as a foundation for the development of composite indicators for the assessment of hospital performance
(Iversen et al., 2012)	2012	Norway	cancer patients	14,227	Cancer Patient Experience Questionnaire (CPEQ)	all cancer types	It can serve as an indicator for the national health quality assessment, helping patients make better decisions and raising the standard of care.
(Karabatić et al., 2022)	2022	Croatia	cancer patients	2460	National Cancer Patient Experience Survey (NCPES)	all cancer types	Highlighting issues that need to be resolved in the Croatian healthcare system for cancer patients
(Moens et al., 2022)	2022	Belgium	Pancreatic Cancer patients	—	Patient Reported Experience Measure for Pancreatic Cancer Care (PREPARE)	Pancreatic Cancer	Encourage people with pancreatic cancer to contribute to disseminating the research's findings to other patient populations.
(Nartey et al., 2022)	2022	UK	lung cancer patients	15,967	National Cancer Patient Experience Survey (NCPES)	all cancer types	Providing information for the creation of cancer Patient-reported Experience evaluation instruments specific to various organs and for patient-centered interventions
(Pham et al., 2019)	2019	UK	cancer patients	18,590	National Cancer Patient Experience Survey (NCPES)	all cancer types	It is helpful to track and assess different therapies by looking at whether tumor staging and diagnostic paths for cancer patients are independently related to evaluating variations in the experience that cancer patients report.
(Reid et al., 2023)	2023	Australia	cancer patients undergoing chemotherapy and or radiation therapy	684	Patient-reported Experience Measure Cancer (PREM-C)	all cancer types	Used in healthcare settings to guide safety and quality improvements, as well as to gauge the level of patient-centeredness
(Roth et al., 2020)	2020	USA	cancer patients who received chemo–therapy	2304	Hospital Consumer Assessment of Healthcare Providers and Systems (HCAHPS)	all cancer types	Increased patient participation in disease management
(Rudolph et al., 2019)	2019	Germany	Patients with colorectal and breast cancer	245	The Danish National Cancer Patient Questionnaire (The German adaptation)	all cancer types	German translation of the Danish National Cancer Patient-reported Experience Questionnaire was used for the feasibility study.
(Saunders et al., 2014)	2014	UK	cancer patients	69,086	National Cancer Patient Experience Survey (NCPES)	all cancer types	To meet patient expectations and enhance the standard of care, encourage patients to take a more active role in controlling their conditions.
(Taibi et al., 2023)	2023	France	patients with primary or secondary peritoneal surface malignancies (PSMs)	—	A core set of patient-reported outcomes and patient-reported experience measures for peritoneal surface malignancies (COMETE)	PSMs	Improve symptom management and avoid untoward occurrences using Patient-reported Experience data to make patients more tolerant of chemotherapy or surgery.
(Wagland et al., 2017)	2017	UK	cancer patients	2992	National Cancer Patient Experience Survey (NCPES)	all cancer types	Examining variations in Patient-reported Experiences from patients with primary known metastases in the malignancy Patient Experience Survey versus individuals with primary unknown malignancy
(Watanabe et al., 2021)	2021	Japan	cancer patients	20,488	Social Experience of Care and Social Life of Patients with Cancer	all cancer types	Establish standards to assess the performance of the country's healthcare system, helping to address the areas where the program needs to be improved.
(Yu et al., 2018)	2018	Taiwan	cancer outpatients	4000	Taiwan versions of the Cancer Patient Experience Survey	all cancer types	It can serve as a foundation for developing a cancer patient-centered care model.
(Shah et al., 2024)	2024	UK	bladder cancer patients		National Cancer Patient Experience Survey(NCPES)	all cancer types	Examining How Treatment Type and Patient Factors Affect Patient Experience Measures
(van Hof et al., 2024)	2024	Netherlands	cancer outpatients	—	PREM-item bank	all cancer types	Construct a flexible, treatment-focused patient experience assessment system.

### 3.3 Listing of patient-reported experience measures assessment tools for cancer patients

The Patient-Reported Experience Measures assessment tools are listed according to name, country, development agency, dimension, questionnaire entry, etc. If there are different versions of the same assessment tool, the latest version is selected. The 11 assessment tools are: the Hospital Consumer Assessment of Healthcare Providers and Systems (HCAHPS) (Roth et al., 2020; Fauer et al., 2021; Cha et al., 2022), the National Cancer Patient Experience Survey (NCPES) (Saunders et al., 2014; Clucas, 2016; Wagland et al., 2017; Yu et al., 2018; Alessy et al., 2019; Pham et al., 2019; Brookes and Baker, 2022; Gomez-Cano et al., 2022; Karabatić et al., 2022; Nartey et al., 2022; Arditi et al., 2023), the Cancer Patient Experience Questionnaire (CPEQ) (Iversen et al., 2012), the Patient-Reported Experience Measure for Cancer (PREM-C) (Reid et al., 2023), the Danish National Cancer Patient Questionnaire (Rudolph et al., 2019), the Social Experience of Care and Social Life of Patients with Cancer (Watanabe et al., 2021), the Core Set of Patient-Reported Outcomes and Experience Measures for Peritoneal Surface Malignancies (COMETE) (Taibi et al., 2023), the Patient Reported Experience Measure for Pancreatic Cancer Care (PREPARE) (Moens et al., 2022), the Experienced Patient-Centeredness Questionnaire (EPAT) (Christalle et al., 2022), the LifeCourse Experience Tool (Fernstrom et al., 2016) and the PREM-item Bank (van Hof et al., 2024).

The Hospital Consumer Assessment of Healthcare Providers and Systems (HCAHPS) cancer patient version, developed by the American Institutes for Research and the Mayo Clinic (CAHPS Cancer Care Survey: Cancer Surgery, no date), includes three parallel instruments for radiation oncology, medical oncology, and cancer surgery, primarily used for assessing cancer patient experiences and satisfaction. The National Cancer Patient Experience Survey (NCPES), based on the NHS Patient Experience Framework, covers various aspects of the cancer patient experience, including GP visits, inpatient care, and outpatient follow-up, to gather recommendations for improving patient care (Karabatić et al., 2022). Established in 2004 by the Norwegian Centre for Health Services Research, the Cancer Patient Experience Questionnaire (CPEQ) can be administered to adult cancer patients across various settings, including inpatient hospitals and outpatient clinics (Pettersen et al., 2004). The Patient-Reported Experience Measure for Cancer (PREM-C) is grounded in a six-domain patient-centered framework, aimed at measuring patient-centered care and guiding quality improvement (Reid et al., 2023). The Danish National Cancer Patient Questionnaire (Sandager et al., 2015) consists of two-thirds Patient-Reported Experience Measure and one-third Patient-reported Outcome Measures and assesses patient experiences from the onset of symptoms to discharge. Created by the National Cancer Center of Japan (Watanabe et al., 2021), the Social Experience of Care and Social Life of Patients with Cancer focuses on the chronological experience of patients before, during, and after treatment. Core Set of Patient-Reported Outcomes and Experience Measures for Peritoneal Surface Malignancies (COMETE) (Taibi et al., 2023) specifically addresses the information needs of patients with peritoneal surface malignancies regarding diagnosis, therapy, and complications. The Patient Reported Experience Measure for Pancreatic Cancer Care (PREPARE) tool (Moens et al., 2022), guided by the MRC framework, measures the needs and experiences of pancreatic cancer patients throughout their care trajectory, from diagnosis to follow-up. The Experienced Patient-Centeredness Questionnaire (EPAT) (Christalle et al., 2022) evaluates the level of patient-centeredness for patients receiving chronic disease care in Germany, applicable in both inpatient and outpatient settings. The LifeCourse Experience tool (Fernstrom et al., 2016) is based on the principle of "know me, ask me, listen to me, hear me, guide me, respect me, comfort me, support me," aimed at examining experiences of individuals with major chronic illnesses. To dynamically assess the quality of care for outpatient cancer patients, Dutch scholar van Hof (van Hof et al., 2024) developed the PREM-item Bank through literature reviews, focus group analyses, qualitative assessments, and quantitative selection.

### 3.4 Evaluation system for patient-reported experience measures in cancer patients

Seven papers on the development and validation of evaluation instruments for Patient-Reported Experience Measures in cancer patients were included in the final collection. The development of these instruments typically follows a phased approach, which includes creating an initial item pool, conducting pilot testing, and implementing psychometric validation. Each evaluation tool is based on a multifaceted framework, with questionnaires using Likert scale ratings. The number of items ranges from 15 to 157, divided into 5 to 16 distinct domains (i.e., categories). Five of the included Patient-Reported Experience Measures evaluation tools underwent reliability and validity testing, with Cronbach's alpha coefficients ranging from 0.70 to 0.91 (Pettersen et al., 2004; Fernstrom et al., 2016; Karabatić et al., 2022; Reid et al., 2023; CAHPS Cancer Care Survey: Cancer Surgery, no date). The remaining six tools have yet to undergo this testing.

The dimensions and content of the assessment tools focused on patient perceptions during consultations. Commonly addressed topics included health education and information sharing, service integration, transition and continuity of care, patient respect, emotional support, and the involvement of family and friends. Two of the instruments were developed using established theoretical frameworks (Karabatić et al., 2022; Moens et al., 2022): the National Health Service (NHS) Patient Experience Framework and the Medical Research Council (MRC) Framework. Four of the tools are still in the development and testing phases, with reliability and validity yet to be confirmed (Sandager et al., 2015; Christalle et al., 2022; Taibi et al., 2023; van Hof et al., 2024). For more details, see Table 3.

**Table 3:** Patient-Reported Experience Measures Assessment Tools for Cancer Patients (by tool name).

Type	Assessment tool	Author, country, Year	Main themes	Reliability	Description
Generic	Hospital Consumer Assessment of Healthcare Providers and Systems (HCAHPS) (CAHPS Cancer Care Survey: Cancer Surgery, no date)	American Institutes for Research and Mayo Clinic, USA, 2016	Includes 26 entries covering five topics: patient self-management (4 entries), emotional communication (7 entries), visits (8 entries), shared decision-making (3 entries), and one entry on the hospital's overall assessment.	Cronbach's α=0.88	A cancer care version of the HCAHPS was developed based on the HCAHPS, which is mainly used for cancer patient experience surveys and patient satisfaction measurements to improve the quality of healthcare services. Still, it does not address the dimension of accessibility to hospital admissions, and the collection of information during the survey may not be comprehensive enough.
	National Cancer Patient Experience Survey (NCPES) (Karabatić et al., 2022)	National Health Service (NHS), UK, 2010	There are sixteen categories in all, including family doctor, illness diagnosis, issues, selecting the best course of treatment, clinical nurse specialists, cancer support, surgery, hospital doctors, ward nurses, hospital care and treatment, information access before discharge, home support, hospital care for day/outpatients, on-time appointments, general medical care, holistic NHS care, and more.	Cronbach's α=0.83	The NCPES was created based on patients in Western countries and may not apply to cancer patients in Asian countries. Additionally, excessive survey items may lead to inaccurate content completion. Despite being developed based on the NHS Patient Experience Framework, the dimension content is more comprehensive.
	Cancer Patient Experience Questionnaire (CPEQ) (Pettersen et al., 2004)	Norwegian Center for Health Services Research, Norway, 2004	With 127 entries spread across ten categories, the majority of the questionnaire's responses center on the following topics: hospital surroundings and amenities, patient safety, doctor and nurse services, information sharing, complaint details, prescription information, family interactions, structure, and general satisfaction	The retest correlation coefficient is between 0.57 and 0.85, and the Cronbach alpha coefficient satisfies the 0.70 requirement.	The CPEQ, which comes in an inpatient and an outpatient version, emphasizes usability and practicality while offering sufficient data relevant to patients in most medical and surgical wards. Despite this, the questionnaire contains many items, some of which are only relevant to confident respondents.
	Patient-reported Experience Measure Cancer (PREM-C) (Reid et al., 2023)	Carol Reid, Australia, 2023	The eight dimensions are respect for patient values, preferences, and expressed requirements; physical and emotional comfort; support from family and friends; coordination and holistic care; communication of information and health education; and access to resources.	Cronbach's alpha coefficients for each dimension ranged between 0.8 and 0.9	The questionnaire can be used to evaluate patient-centered care because it was created using a strict psychometric test methodology. However, it is still in the early stages of research and needs more validation regarding reliability and validity in other cancer patients and settings.
	Danish National Cancer Patient Questionnaire (Sandager et al., 2015)	Danish Cancer Society, Denmark, 2010	The questionnaire included contact with GP during diagnosis, waiting time for consultation, coordination and continuity of care, information, and communication, patient and relative involvement, continuity of information, and continuity of coordinators and individuals, as well as three additional qualitative comment entries: Qualitative comments on diagnosis, qualitative comments on patient and family participation, and qualitative comments on continuity and responsibility.	—	Although the questionnaire is thorough and a significant step in bettering patients' experiences with cancer care, it is not analytically statistically tested, which makes it less generalizable, and there are too many items to guarantee response rates and the generalizability of results.
	Social Experience of Care and Social Life of Patients with Cancer (Watanabe et al., 2021)	National Cancer Center of Japan, Japan, 2015	The questionnaire addresses the following topics: social life before and after treatment, treatment choice, and cancer diagnosis.	—	The questionnaire can be used to evaluate cancer patients' experiences before, during, and following treatment; however, allowing family members and other people to report on behalf of the patient may result in an underestimation of the patient's experiences.
	PREM-item bank (van Hof et al., 2024)	van Hof, Netherlands, 2024	Eight subjects are covered in the content: healthcare organizations, healthcare professionals' competencies, communication, information and services, patient empowerment, continuity and informal care, and technology and the environment.	—	The item bank applies to all types of cancer and can be used to dynamically evaluate patients' experiences getting cancer care in an outpatient context; however, it has not yet undergone psychometric validation.
Cancer-specific	A core set of Patient-reported Outcomes and Patient-reported Experience measures for peritoneal surface malignancies (COMETE) (Taibi et al., 2023)	Abdelkader Taibi, France, 2023	Contents include feeling satisfied with the care process, how cancer was explained, details about diagnosis, treatment, and complications, how the healthcare team recognized the patient's importance, how the hospital was contacted, and hospital or medical staff contact information.	—	COMETE, which focuses on information about diagnosis, treatment, and complications in PSM patients, was the first to create a core set for patients with particular malignancies. However, the questionnaire has not been externally validated in clinical or research settings.
	Patient Reported Experience Measure for Pancreatic Cancer Care (PREPARE) (Moens et al., 2022)	Belgian Cancer Center, Belgium, 2022	Assessing the requirements and encounters of patients with pancreatic cancer throughout their treatment (from diagnosis to follow-up)	—	The PREPARE questionnaire is presently undergoing development and validation. It uses the MRC framework as a guide to measure the needs and experiences of pancreatic cancer patients across the continuum of care.
	Experienced Patient-Centeredness Questionnaire (EPAT) (Christalle et al., 2022)	Eva Christalle MSc, Germany, 2021	The questionnaire addresses mental health support, essential characteristics of a clinician, the patient-physician relationship, the patient as a person, integration of healthcare and care, teamwork and team building, access to care, coordination and continuity of care, patient information, patient engagement, support from family and friends, good care planning, and emotional support.	—	EPAT evaluates the patient experience from the patient's perspective in terms of the degree of patient-centeredness, primarily for patients with chronic conditions (cancer, cardiovascular diseases, mental disorders, musculoskeletal disorders) in Germany, and is now undergoing a psychometric test.
	LifeCourse experience tool (Fernstrom et al., 2016)	Karl M. Fernstrom USA, 2016	"Care team, communication, and goals of care" has three aspects and 25 entries.	Cronbach's α=0.91	The scale was designed to assess the experiences of people with significant chronic illnesses (heart failure, cancer, or dementia), and the entries are straightforward to create; nevertheless, the item wording and topics are more similar so that factor loadings may be artificially exaggerated.

### 3.5 The application of patient-reported experience measures in cancer patients

The application of Patient-Reported Experience Measures in cancer care encompasses four key areas: (1) Gathering information to develop new therapies or interventions: Patient-Reported Experience Measures are used to understand variations in patient experiences across different patient populations, targeting interventions for improvement (Brookes and Baker, 2022; Arditi et al., 2023). They help assess the proportion of patients providing feedback on both positive and negative experiences and identify key response themes driving evaluations (Clucas, 2016; Christalle et al., 2022). (Brookes and Baker, 2022; Arditi et al., 2023) Patient-Reported Experience Measures are crucial for assessing quality improvement in routine clinical settings (inpatient and outpatient), explaining differences in experience between patient groups, and improving treatment phases based on patient feedback to inform clinical practice (Iversen et al., 2012; Karabatić et al., 2022). Additionally, these findings serve as national quality indicators, providing patients with informed choices and enhancing healthcare delivery (Nartey et al., 2022; Reid et al., 2023). Patient-Reported Experience Measures also highlight areas within oncology care that require improvement and are used to measure the level of patient-centeredness, influencing clinical safety and quality improvement efforts (Yu et al., 2018). (2) Assisting healthcare professionals in monitoring and evaluating therapeutic interventions (Clucas, 2016; Arditi et al., 2023): Patient-Reported Experience Measures allow professionals to examine whether cancer diagnosis pathways and tumor stages are independently associated with variations in patient-reported experiences. These insights help monitor and evaluate the effectiveness of therapies, improve symptom control, minimize adverse events, and assess the efficacy of surgical or chemotherapeutic treatments. (3) Enhancing patient self-management and participation in disease management (Alessy et al., 2019; Arditi et al., 2023): By understanding the experiences of cancer patients undergoing outpatient chemotherapy, Patient-Reported Experience Measures contribute to improving patient involvement in managing their disease. They help gauge patient-reported expertise, enabling the alignment of care with patient expectations and enhancing the overall quality of care. (4) Contributing to performance assessment for national or regional health policy (Watanabe et al., 2021): Patient-Reported Experience Measures provide vital insights for the development, monitoring, and improvement of cancer policies (Cha et al., 2022; Arditi et al., 2023). They support national health policy formulation, contribute to the evidence base driving clinical practice standards, and serve as a foundation for composite metrics that inform hospital performance evaluations (Fernstrom et al., 2016; Gomez-Cano et al., 2022). Moreover, Patient-Reported Experience Measures offer benchmarks for measuring healthcare system performance and identifying areas for improvement in healthcare programs (Watanabe et al., 2021).

## 4 Discussion

### 4.1 Research on patient-reported experience measures in cancer patients is still in its early stages and unevenly distributed across time, regions, populations, and study designs

The 24 selected papers exhibit an upward trend in publication frequency, with one paper published in 2012, 2014, and 2018, two in 2019 and 2021, and seven in 2022. Geographically, the United Kingdom leads with nine publications, followed by the United States with four. Most research participants were patients with breast, lung, prostate, or rectal cancers. Studies employed mixed-methods, qualitative, and quantitative approaches. Much of the literature focuses on the development, testing, and implementation of instruments to measure cancer patients' reported experiences (van Hof et al., 2024), the creation of interventions based on these findings, and the validation of those interventions in clinical practice (Karabatić et al., 2022). Some studies also examine the needs and perspectives of cancer patients to assess the extent to which hospitals are patient-centered, from the patients' viewpoint (Christalle et al., 2022). Patient-Reported Experience Measures provide valuable insights into the emotional and experiential states of patients, offering richer data than traditional quality-of-life ratings. They yield more comprehensive and accurate information compared to survival measures. In light of this, there is a need for the development of high-quality, specialized assessment tools tailored to the unique characteristics and experiences of cancer patients, guided by robust scientific theory.

### 4.2 Patient-reported experience measures assessment tools for cancer patients vary widely, and further validation is needed

Currently, a variety of assessment instruments are available to evaluate Patient-Reported Experience Measures in cancer patients, including computerized tools, scales, and questionnaires, each with unique features and evaluation methods. Selecting the most appropriate tool depends on the study's objectives, target population, focus of the measurement, and patients' health status. In this review, eleven different instruments were identified, but only five had undergone rigorous testing for reliability and validity (Iversen et al., 2012; Fernstrom et al., 2016; Roth et al., 2020; Karabatić et al., 2022; Reid et al., 2023). Furthermore, several assessment tools have not seen widespread use post-development, and their effectiveness remains unclear.

For example, while the Patient-Reported Experience Measure for Cancer (PREM-C) was developed using a rigorous psychometric testing approach, it remains in the early stages of research, and its reliability and validity in other cancer populations and settings need further confirmation (Reid et al., 2023). The Danish National Cancer Patient Questionnaire (Sandager et al., 2015) offers a comprehensive approach to improving cancer care experiences. However, its extensive length poses challenges to ensuring high response rates and the generalizability of results. Additionally, it has not undergone statistical analysis, limiting its broader applicability.

The Core Set of Patient-Reported Outcomes and Experience Measures for Peritoneal Surface Malignancies (COMETE) tool also lacks external validity (Taibi et al., 2023), making it difficult to use in clinical or research settings, while Patient Reported Experience Measure for Pancreatic Cancer Care (PREPARE), developed by the Belgian Cancer Center (Moens et al., 2022), is still undergoing development and validation. Experienced Patient-Centeredness Questionnaire (EPAT) (Christalle et al., 2022) focuses on patients with chronic conditions, including cancer, cardiovascular diseases, mental disorders, and musculoskeletal disorders, and is currently being tested for psychometric robustness. Similarly, the LifeCourse experience tool (Fernstrom et al., 2016) has simple entries, but the similarity in item phrasing and themes could inflate factor loadings, raising concerns about its accuracy. The Dutch PREM-item bank (van Hof et al., 2024) dynamically assesses outpatient cancer care experiences across all cancer types, but it has yet to undergo psychometric validation.

Notably, there is no version of a widely accepted Patient-Reported Experience Measures assessment tool for cancer patients, underscoring the need for cultural adaptation when using such instruments in different contexts. Researchers must validate these tools across multiple centers with large sample sizes to ensure their applicability and effectiveness.

### 4.3 Specific patient-reported experience measures must be developed to achieve greater measurement precision and responsiveness

There remains a lack of specialized assessment instruments tailored to capture the specific experiences of cancer patients. The first core set of Patient-Reported Experience Measures designed specifically for cancer patients is the Core Set of Patient-Reported Outcomes and Experience Measures for Peritoneal Surface Malignancies (COMETE) (Taibi et al., 2023), which focuses on patients with PSM. Similarly, the Belgian Cancer Center began developing Patient Reported Experience Measure for Pancreatic Cancer Care (PREPARE) in 2022, which targets the needs and experiences of pancreatic cancer patients across their care continuum, from diagnosis to follow-up (Moens et al., 2022). Additionally, broader tools like the Patient Experience Tool for Chronic and Serious Diseases (Fernstrom et al., 2016) and the patient-centered Experienced Patient-Centeredness Questionnaire (EPAT) questionnaire (Christalle et al., 2022) cater to patients with various chronic illnesses, including cancer.

However, most of the current Patient-Reported Experience Measures used for cancer patients are universal scales, designed for use across various cancer types (Yu et al., 2018). Several studies have shown that universal Patient-Reported Experience Measures make it difficult to identify specific actions needed to improve care for particular cancer types, as some assessment items may not be suitable for specific conditions (van Hof et al., 2024). Moreover, these generic tools may include irrelevant information and overlook critical aspects of the patient's specific experience, leading to incomplete assessments and potentially leaving patients feeling undervalued (Berkowitz, 2016). Therefore, the development of high-quality, disease-specific Patient-Reported Experience Measures assessment tools is essential. These tools should be grounded in scientific theory and tailored to the unique characteristics of different cancer patient populations to ensure accurate and comprehensive evaluations of their care experiences.

### 4.4 The role of patient-reported experience measures in enhancing patient-centered care

Patient-Reported Experience Measures is considered a pillar of high-quality care and play a vital role in advancing patient-centered care (Clucas, 2016; Arditi et al., 2023). Incorporating Patient-Reported Experience Measures results aims to boost patient engagement in disease self-management, improve treatment outcomes, enhance patient safety, support accurate and effective care, and guide patient-centered clinical practice (Saunders et al., 2014; Reid et al., 2023). Early research suggests that implementing Patient-Reported Experience Measures in everyday nursing practice can help providers recognize where change is needed and galvanize transformation (Wild et al., 2024). However, implementing these principles is challenging and requires the collaboration of multiple stakeholders, including patients, healthcare providers, and administrators (Verma and Desai, 2024). Despite the policy focus on Patient-Reported Experience Measures, there are currently few published regarding their impact on clinical practice. Evidence of whether Patient-Reported Experience Measures improve quality of care or support person-centered care in the clinical setting remains largely theoretical. While the patient-centered care model is highly suited for addressing the needs of cancer patients, much like other chronic diseases, further research is necessary to optimize its application and fully realize its benefits in oncology.

### 4.5 Strengths and limitations

This scoping review offers several strengths. First, to minimize the risk of omitting relevant studies, we focused on the evolution of Patient-Reported Experience Measures evaluation tools over the past decadesand ensured that the literature review was updated accordingly. Additionally, we performed a pre-search to refine and adjust the research plan and search strategy based on the initial findings, ensuring purposefully and logically. Importantly, the review followed the PRISMA-ScR guidelines rigorously, which enhances the scientific rigor and standardization of the work.

However, there are limitations to this study that must be acknowledged. First, while we searched three widely-used English-language databases to balance efficiency and workload, we did not include non-English literature, which may have led to the exclusion of relevant studies published in other languages. Second, this review is limited to already pulished publications. As some studies do not provide complete or updated versions of Patient-Reported Experience Measures, and certain clinical applications of Patient-Reported Experience Measures may remain unpublished, our findings could potentially underestimate the current usage of Patient-Reported Experience Measures in routine healthcare practice.

## 5 Conclusion

This paper presents the current state of research on Patient-Reported Experience Measures in cancer patients through a scoping review, highlighting that the field is still in its early stages and lacks uniform development. While existing assessment tools cover a broad range of content, the effectiveness and validity of several instruments require further investigation. The majority of the current evaluation techniques rely on universal scales, with a notable scarcity of high-quality, tailored instruments specifically designed for cancer patients. Future research should focus on improving and validating these tools by employing more robust study designs and contextual applications. Such studies should be grounded in the unique healthcare experiences of cancer patients, drawing on scientific theoretical frameworks, comprehensive literature reviews, and rigorous analysis. The development of high-quality, specific Patient-Reported Experience Measures tailored to cancer patients is essential to capture the nuances of their healthcare experiences.

These improved tools can then be used to investigate current trends and influencing factors in cancer care, allowing for the creation of targeted intervention programs that enhance healthcare services and patient-centered care practices. Moreover, these instruments can serve as a theoretical foundation for improving patient-physician communication, identifying potential healthcare system shortcomings, and ultimately improving both the quality of care and the quality of life for cancer patients.

## Funding

This work was supported by the 10.13039/501100001809National Natural Science Foundation of China (Grant No. 82173627). The funders had no role in considering the study design or in the collection, analysis, interpretation of data, writing of the report, or decision to submit the article for publication.

## Ethics statement

Not required.

## Appendix A. Supplementary data

Supplementary data related to this article can be found at https://doi.10.17605/OSF.IO/RVTQG

## CRediT authorship contribution statement

**Qiongjie Shao:** Writing – review & editing, Writing – original draft, Data curation. **Wei Zhang:** Writing – review & editing, Data curation. **Hongjuan Lang:** Methodology, Conceptualization. **Yan Wang:** Methodology, Conceptualization. **Han Tang:** Methodology, Conceptualization. **Juan Du:** Software, Data curation. **Ying Liang:** Software, Data curation. **Pengyu Jing:** Software, Data curation. **Zhongping Gu:** Software, Data curation. **Xiaolong Yan:** Writing – review & editing. **Lei Shang:** Writing – review & editing.

## Declaration of competing interest

The authors declare no conflict of interest.
